# Standardizing upper arm movement definitions across observational and sensor-based methods: A Delphi consensus study among European ergonomics experts

**DOI:** 10.5271/sjweh.4288

**Published:** 2026-05-01

**Authors:** Anders Dreyer Frost, Mikael Forsman, Luiz Augusto Brusaca, Andreas Holtermann, Lars Louis Andersen, Karen Søgaard, Nidhi Gupta

**Affiliations:** 1 National Research Centre for the Working Environment, Copenhagen, Denmark.; 2 Department of Sports Science and Clinical Biomechanics, University of Southern Denmark, Odense, Denmark.; 3 Division of Ergonomics, KTH Royal Institute of Technology, Stockholm, Sweden.; 4 Institute of Environmental Medicine, Karolinska Institutet, Stockholm, Sweden.; 5 Department of Occupational Health, Psychology and Sports Sciences, University of Gävle, Gävle, Sweden.

**Keywords:** delphi technique, elevated arm work, ergonomic assessment, static posture

## Abstract

**Objectives:**

Musculoskeletal disorders from repetitive upper arm movements contribute substantially to sickness absence and productivity loss. Despite widespread use of observational and sensor-based assessments, inconsistent definitions hinder comparison across studies and translation to practice. This study explored threshold criteria for defining upper arm movements and static postures across observational and sensor-based approaches and examined conceptual differences between practical observability and biomechanical measurability.

**Methods:**

We conducted a two-round Delphi study following the ACCORD guidelines. We invited 35 European experts to rate agreement on proposed definitions. A consensus criterion was set to ≥75% agreement. A thematic analysis of free-text responses guided definition revisions between rounds.

**Results:**

Fifteen (43%) and fourteen (93% retention) completed rounds 1 and 2, respectively. Consensus defined a fast-paced movement as ≤1 second (80% agreement) and static posture as ≥4 seconds with ±5° movements (87% agreement). No agreement emerged regarding the minimum amplitude threshold for defining an arm movement (eg, 10° versus 20°; 53% agreement). Experts’ comments reflected a tension between observability, favoring higher amplitude thresholds, and biomechanical relevance, favoring lower thresholds, while highlighting velocity’s importance.

**Conclusions:**

Expert consensus on time-based thresholds for fast-paced movements and static postures provides a starting point for standardized ergonomic assessment. The absence of consensus on amplitude thresholds highlights the need for field validation studies examining which thresholds capture measurement reliability and prediction of musculoskeletal health outcomes. These findings support efforts toward transparency and alignment in upper arm exposure definitions across research and practice, while acknowledging remaining conceptual and methodological challenges.

Musculoskeletal disorders (MSD) impose a substantial burden on individuals, workplaces, and society. In Central Europe, MSD accounted for over 260 000 years lived with disability in 2020, representing a 47% increase in age-standardized rates since 1990 (1, 2). Among MSD, neck and shoulder disorders represent a particularly costly subset. In Denmark, neck pain results in nearly 2.4 billion euros in annual production loss due to sickness absence and premature death (3), to which physically demanding work contributes substantially (4).

Despite strong evidence linking upper arm movements to the above-mentioned outcomes, fundamental inconsistencies in how we define and measure arm movements undermine translation of research into effective workplace prevention strategies.

Several exposure characteristics, such as accumulated time with the upper arm elevated >60° or >90° (4–6), median generalized velocity of ≥60°/s over a full working period (7), repetitive movements (8–10), and forceful exertions (8, 11, 12), have been associated with an increased risk of shoulder pain and long-term sickness absence (13–18).

Traditionally, observational measurement tools have been used to assess upper-arm work in occupational settings. Tools such as the Rapid Upper Limb Assessment (RULA) (19) and the Occupational Repetitive Actions (OCRA) checklist (20) are widely applied due to their simplicity, low cost, and structured approach. However, observational tools rely heavily on subjective judgment, making them prone to inter-rater variability and limited reliability (21–23). More recently, wearable sensor technologies, such as accelerometers and inertial measurement units (IMUs), have emerged as promising tools that enable more precise and continuous quantification of upper arm elevation, velocity, and repetition over extended periods (24). Although wearable sensor-based measurement tools reduce observer bias and improve measurement precision (25, 26), researchers apply widely varying amplitude and duration thresholds to define movements across both observational and sensor-based studies (27). This methodological heterogeneity prevents comparison of findings across studies, weakens evidence synthesis, and impedes its translation into clear ergonomic guidelines.

Previous research has made several attempts to standardize the assessment of upper-limb exposures, through both observational tools (19–22) and sensor-based measurement tools (24–27). These efforts have typically focused on specific measurement systems or posture categories rather than on the underlying criteria that define when a movement begins and ends. Consequently, no unified or consensus-based framework currently exists to harmonize upper arm movement definitions across methodological approaches. These methodological differences hinder comparison across studies and translation of research into ergonomic guidelines.

Achieving expert consensus on operational definitions of upper-arm movements may improve standardized measurement and translation of research into prevention. When researchers, ergonomists, and occupational health professionals share a common understanding of what counts as “an upper-arm movement”, they can more effectively identify potentially harmful patterns, communicate risks, and design appropriate workplace interventions. Such a consensus may strengthen the preventive potential of ergonomic assessment tools. In addition, this highlights the need for expert-informed, operational definitions of upper-arm movements that can be applied consistently across both observational and sensor-based methods.

To address this critical gap, this Delphi study aimed to explore whether expert consensus could be achieved on operational definitions of upper-arm movements and static-arm postures that are applicable across both observational and sensor-based assessment approaches. Specifically, the study sought to identify threshold criteria related to amplitude and duration and examine where conceptual tensions may arise between practical observability and biomechanical measurability when defining upper-arm movements in occupational ergonomics.

## Methods

### Study design

In this study, we used a modified Delphi method design (28) and reported in accordance with the ACCORD guideline for consensus-based studies (29). The modified Delphi method is an iterative survey approach for achieving expert consensus (30, 31). We chose a priori of the study to conduct at most three rounds of surveys, allowing us to stop after two rounds if responses stabilized, meaning that further rounds would be unlikely to change the outcome (≤5 percentage points change in proportion “agree/strongly agree”) (31). The Delphi study was conducted from September to November 2024. Data were collected using SurveyXact (SurveyXact, Aarhus, Denmark). Microsoft Excel (Microsoft Corporation, Redmond, Washington, USA) was used to store and manage data.

Ethical approval was obtained from the relevant regional research ethics committee (F-24018056). All participants provided informed consent prior to participation, and the study was conducted in accordance with the Declaration of Helsinki.

### Expert panel

Following Delphi method guidelines recommending a homogeneous expert panel size of 10–15 experts (30, 32), we invited 35 European researchers to account for potential dropout and to ensure broad representation.

Eligibility criteria required that the participants worked within occupational research on physical activity, ergonomics, or health effects of upper-arm movements and were currently employed at a European research institution.

**Figure 1:**
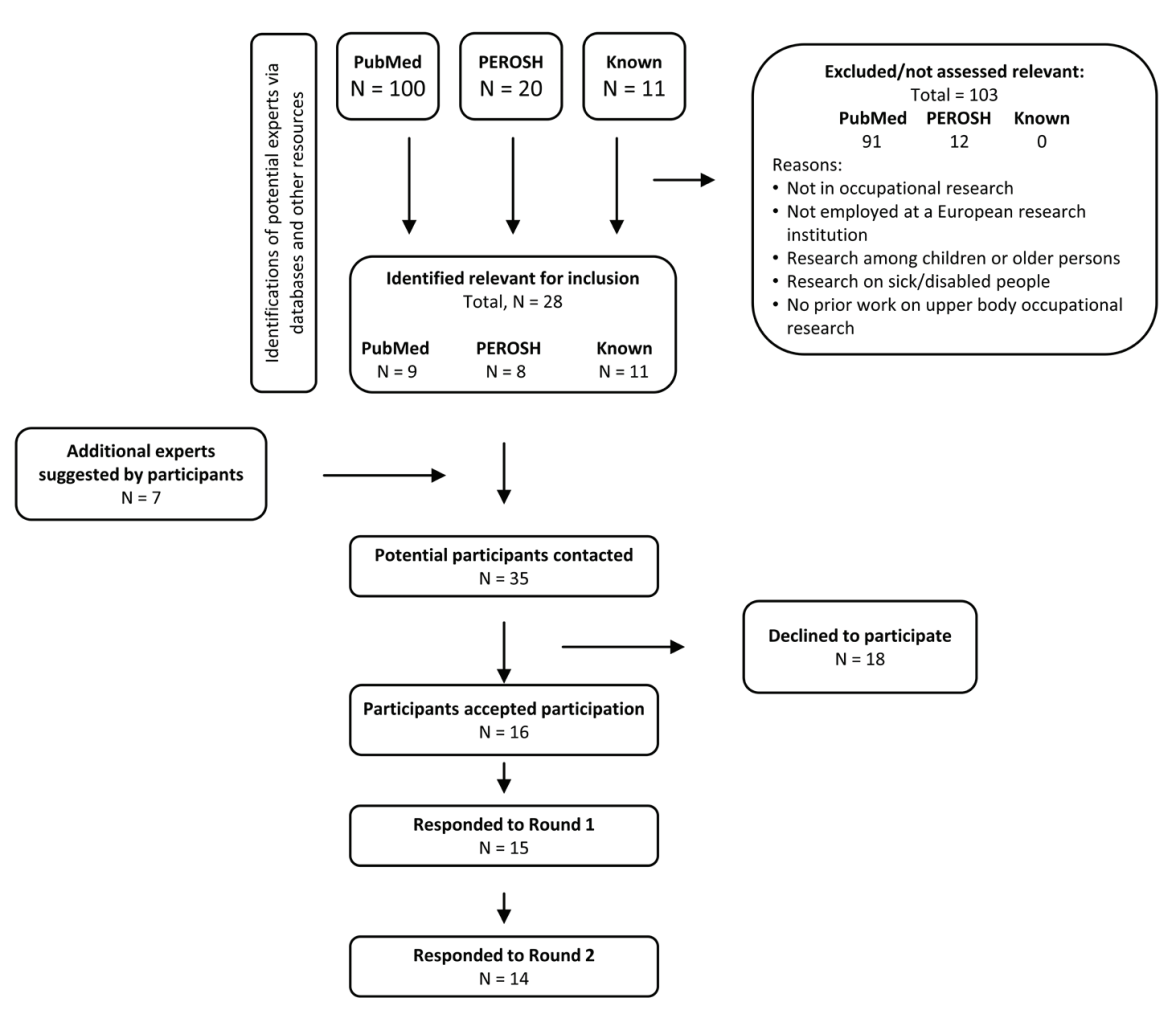
Flowchart of expert selection and Delphi rounds.

Potential experts were identified using four strategies: (i) a literature search (top 100 relevant publications screened; corresponding authors invited); (ii) experts identified through the Partnership for European Research in Occupational Safety and Health (PEROSH) (33); (iii) known experts within the relevant field; and (iv) additional experts suggested by already invited participants and validated by the steering group (snowball method). The authors of this paper did not participate in the expert panel due to their role in conducting the Delphi study. Figure 1 illustrates the identification and inclusion process for expert participants.

### Survey development and pilot testing

The PEROSH report “Assessing Arm Elevation at Work With Technical Systems” (33) was the basis of key terms and definitions. A literature search was conducted in (see supplementary material, www.sjweh.fi/article/4288, appendix A) to identify previously applied definitions of occupational upper-arm movements. The literature search did not identify any prior studies that had formally defined what constitutes one complete upper-arm movement from a technical or biomechanical perspective. Consequently, all definitions were developed through discussion within the author group.

Throughout this paper, the term “arm movement” refers specifically to movements of the upper-arm (humerus). Arm elevation was operationalized relative to the gravitational vertical, consistent with established technical measurement approaches (33). This reference was chosen to ensure compatibility with inclinometer- and accelerometer-based assessments, which quantify arm elevation relative to gravity. Using the vertical as a reference reduces ambiguity related to trunk movement and facilitates comparability across observational and sensor-based methods.

To facilitate expert panel comprehension, each proposed definition in round 1 (R1) of the Delphi survey was supplemented with illustrative figures and short videos. The R1 survey was pilot tested internally with members of the author group (N=6) to assess the readability of the definitions and refine the survey structure and clarity of the instructions. Minor revisions were made to the wording of instructions, while the definitions themselves remained unchanged (30, 32, 34).

Each Delphi round was open for a period of two weeks, with a single reminder email sent after one week.

The experts were asked to rate their agreement with each definition on a five-point Likert scale, with the following options: (i) strongly disagree, (ii) disagree, (iii) neither agree or disagree, (iv) agree, (v) strongly agree. Open-ended fields were included to allow experts to provide comments or propose alternative formulations. Consensus was defined a priori as ≥75 percentage of panel members selecting “agree” or “strongly agree”, a level considered optimal for rating statements in Delphi studies (30, 34–36). If consensus was not reached in a given round, a modified definition was presented in the subsequent round for re-evaluation.

### Round 1 survey

The R1 survey included four sections: (i) background information (age, gender, experience, position, degree); (ii) proposed definitions of a minimum observable arm movement (amplitude required for a movement to be counted); (iii) definitions of a fast-paced arm movement (maximum duration of one movement); and (iv) definitions of a static-arm posture (minimum duration and amplitude required for a posture to be classified as static). Each item was accompanied by figures and short videos to aid comprehension, and experts could provide free-text comments. Definitions that reached consensus were excluded from subsequent rounds. The arm movement definitions, fast-paced, and static postures were framed from an observational perspective.

Detailed information about the original survey presented in R1 is provided in supplementary appendix B. The full wording of all definitions presented in R1 is provided in table 1.

**Table 1:** Definitions of arm movements presented to the expert panel in rounds 1 and 2 of the Delphi survey.

Definition category	Key criterion
Minimum observable arm movement	An arm movement starts when the arm angle exceeds a predefined amplitude threshold (θ) above a previous local minimum and ends when the arm angle decreases by at least θ from a local maximum, reaching a subsequent local minimum. **Tested amplitude thresholds:** 10°, 20°, and 50°.
Fast-paced arm movement	An arm movement is considered fast-paced when the entire movement is completed within ≤t seconds. **Tested duration thresholds (t):** ≤1 second, ≤2 seconds, and ≤3 seconds.
Static arm posture	The upper arm is raised to a peak angle and maintained for ≥4 seconds within an angular deviation of ±θ. Minor arm movements within this range do not terminate the static posture. Tested angular deviations (θ): ±5°, ±10°, and ±15°.
Sensor-based arm movement	An upper arm movement starts at a local minimum when the arm angle exceeds a predefined amplitude threshold (θ) and ends when the arm angle decreases by at least θ from a local maximum, reaching a subsequent local minimum. Tested amplitude threshold: 5°.

### Round 2 survey

The R2 survey built directly on that of R1, based on the answers from both the quantitative summaries and qualitative comments. The R2 survey consisted of two sections. In section one, the experts were presented with revised definitions of a minimum observable arm movement, updated based on feedback from R1 and subsequent discussions within the steering group. For example, clarifications to amplitude thresholds were made for consistency and improved clarity. In section two, we added one new measurement component based on participant suggestions, concerning a definition of a minimum arm movement using wearable sensors.

Summaries of the quantitative R1 results, including numbers and percentages, were presented to experts in a table format. Free-text answers were categorized into themes, and changes to definitions and examples were explicitly marked for transparency. The experts were asked to reconsider their responses in light of the group feedback to encourage convergence towards consensus. The full wording of the revised and new definitions presented in R2 is provided in table 1.

### Analytical approach and questionnaire adjustments

*Quantitative analysis.* The responses were pseudonymized to enable tracking across rounds while maintaining confidentiality. After each round, the number and proportion of experts selecting each of the five Likert-scale categories were calculated. Based on this, the steering group reviewed the proposed definitions.

*Qualitative analysis.* One author initially coded all free-text comments using an inductive thematic approach (37). First, they systematically reviewed and coded all open-ended responses from R1, identifying key concepts and grouping similar statements into preliminary thematic categories. This procedure was repeated for responses collected in R2. The systematic procedure consisted of iteratively reading all responses, assigning descriptive labels to recurring ideas, and merging overlapping codes into broader categories. The preliminary thematic groupings were subsequently discussed within the author group, which decided, based on these results, to retain only one definition for observable movements and to introduce an additional definition for sensor-based measurements in R2. These adjustments ensured that expert feedback was appropriately reflected in the subsequent survey round. Inter-coder reliability statistics were not calculated, as the qualitative analysis was conducted to inform refinement of the survey definitions rather than to produce standalone qualitative findings.

## Results

### Summary of the Delphi rounds and results

A total of 131 potential experts were screened for eligibility. Of these, 103 were excluded because the identified studies did not focus on either occupational arm movements, targeted children or patient populations, or the experts were not employed at a European research institution. The remaining 28 experts met the inclusion criteria and were invited to participate. Invited experts nominated an additional 7 experts, so 35 experts were invited to answer the Delphi survey. In total, 16 experts (46%) accepted the invitation to join the Delphi survey (figure 1). Of these, 15 (43% of those invited) completed R1, and 14 (93% of those who participated in R1) completed R2.

Participant characteristics are presented in table 2. The mean age of the 16 participants who accepted the invitation was 44.7 years, and 69% were male (N=11). Positions included professor/senior researcher (44%), assistant/associate professor or postdoc (19%), and other academic positions such as researcher or PhD student (38%). In addition, 47% of the experts had >16 years research experience. Most participating experts were based in Sweden, Denmark, Germany, and The Netherlands.

**Table 2:** Descriptive characteristics of the invited participants.

		Participants				Non-participants				Total		
		N	%	Mean		N	%	Mean		N	%	Mean
Number of invited experts		16	46			19	54			35	35	
Age				44.7				-			-	-
Sex												
	Female	5	31			9	47			14	40	
	Male	11	69			10	53			21	60	
Position												
	Professor/senior researcher	7	44			7	37			14	40	
	Assistant/associate professor/postdoc	3	19			4	21			7	20	
	Other (researcher, PhD student)	6	38			8	42			14	40	
Years of experience in research												
	6–15	8	53				-			8	53	
	≥16	7	47				-			7	47	
Highest educational degree obtained												
	PhD	13	87				-			13	87	
	Other	2	13				-			2	13	
Country												
	Sweden	6	38			3	16			9	26	
	Denmark	3	19			0	0			3	9	
	Germany	2	19			0	0			2	6	
	The Netherlands	2	13			1	5			3	9	
	Belgium	1	6			1	5			2	6	
	Norway	1	6			3	16			4	11	
	Switzerland	1	6			0	0			1	3	
	Austria	0	0			1	5			1	3	
	Finland	0	0			1	5			1	3	
	France	0	0			1	5			1	3	
	Italy	0	0			2	11			2	6	
	Slovenia	0	0			2	11			2	6	
	Spain	0	0			1	5			1	3	
	United Kingdom	0	0			2	11			2	6	
	Poland	0	0			1	5			1	3	

### Round 1

Table 3 summarizes the R1 responses, which comprised three sections and nine items on arm movement definitions. Consensus was reached for two of the three movement characteristics. For the fast-paced arm movement, 80% of experts agreed that: “An arm movement is considered fast-paced when the whole arm movement is performed within ≤ 1 second” (table 3).

**Table 3:** Consensus ratings for proposed definitions of observable and sensor-based arm movements. **Bold values indicate ≥75% consensus achieved**. [– = the definition was not reassessed in that round].

		Round 1 ^a^			Round 2 ^a^	
		N ^b^	% ^b^		N ^b^	% ^b^
Minimum observable arm movement definitions						
	1 (10°)	8	53		-	-
	2 (20°)	8	53		-	-
	3 (50°/10°)	0	0		7	53
Fast-paced arm movement definitions						
	1 (≤1 second)	**12**	**80**		-	-
	2 (≤2 seconds	8	54		-	-
	3 (≤3 seconds)	0	0		-	-
Static-arm posture definitions						
	1 (≥4 seconds ±5°)	**13**	**87**		-	-
	2 (≥4 seconds ±10°)	7	47		-	-
	3 (≥4 seconds ±15°)	0	0		-	-
Sensor-based minimum arm movement definitions						
	1 (5°)	-	-		9	67

^a^ Retention from round 1 to 2 was 14 of 15 experts (93%). ^b^ Values indicate number (%) of experts responding “agree” or “strongly agree,” relative to the number of experts responding in that round.

For the static-arm posture, 87% agreed that: “An upper arm is raised to a certain peak angle and maintained around this peak for ≥ 4 seconds (T1 threshold), while minor movements (± ≤5°; A1 threshold) do not terminate the posture.”

In contrast, consensus was not reached for the minimum observable arm movement, which included three proposed definitions, based on three amplitude thresholds (10°, 20°, and 50°). None reached the predefined ≥75% consensus threshold: 53% agreed with definitions 1 and 2, and none agreed with definition 3 (table 3).

All participating experts (100%) provided free-text comments on the minimum observable arm movement. Most comments addressed three aspects: (i) which amplitude threshold should define a movement (80% commented on this), (ii) whether dynamic parameters such as angular velocity should be included (13%), and (iii) the practical relevance of examining such movements (40%). The comments reflected the lack of consensus, with several experts emphasizing the need to clarify the most relevant amplitude (eg, arm angle) for defining an observable movement. Experts noted that slower large-amplitude movements are easier to observe, whereas rapid small-amplitude movements may be overlooked. Others questioned the feasibility of applying such definitions in observational settings and suggested that separate definitions could be developed for observational versus sensor-based assessments. Accordingly, R2 included both a revised definition for a minimum observable arm movement (20°) and a new definition tailored to sensor-based measurement (5°) (table 3).

### Round 2

In R2, the survey comprised two sections with two items. The sections on a fast-paced arm movement and a static-arm posture were omitted as consensus had already been reached in R1. Based on the R1 comments, a new section was introduced on defining a minimum arm movement for sensor-based measurements. Detailed information about the R2 survey is provided in supplementary appendix C.

Consensus was not achieved for either the minimum observable arm movement (53% agreement) or the minimum arm movement using sensor-based measurements (67% agreement). All experts (100%) provided free-text comments. These comments mainly addressed the applicability and feasibility of using amplitude-based definitions for guiding observational assessments. Experts highlighted a trade-off between lower thresholds (eg, 10–20°) to capture subtle and potentially relevant movements that are difficult to detect reliably, whereas higher thresholds (eg, 30–50°) are easier to judge, but with a risk of excluding movements that might contribute to musculoskeletal disorders. Several experts emphasized that technically detailed definitions are better suited for implementation with sensors, while observational coding may serve a complementary role.

Although full consensus was not reached, a threshold around 20° was frequently mentioned as a pragmatic compromise. The decision to end the consensus process after R2 was based on the observed stability of expert opinions.

Table 3 presents consensus ratings for all proposed definitions across both rounds, showing the proportion of experts who agreed or strongly agreed with each definition.

## Discussion

This study explored whether expert consensus could be achieved on operational definitions of arm movements and static postures for ergonomic assessment. The Delphi survey was framed around observable arm movements, as reflected in the instructions provided to participants in the first survey round. However, the survey did not require experts to explicitly distinguish between observational feasibility and biomechanical interpretation when evaluating the proposed definitions. Consequently, it cannot be determined whether the observed consensus on duration criteria primarily reflected observational detectability, biomechanical reasoning, or a combination of both.

Consensus was achieved for two definitions: a fast-paced arm movement (≤1 second in duration) and a static-arm posture (maintained for ≥4 seconds within ±5°). For the fast-paced and static definitions, free-text comments primarily concerned the appropriateness of time thresholds and the dependence on movement amplitude and velocity, including whether minor arm movements should terminate a static posture. These time-based thresholds provide operational criteria for categorizing fast-paced movements and static postures in ergonomic assessments.

No consensus was reached on the minimum observable arm movement or the corresponding sensor-based definition. Agreement rates for these items (53% and 67%) remained below the predefined 75% consensus threshold. The experts’ free-text comments shed light on the divergence, with many emphasizing the need to balance practical observability with biomechanical relevance, while others argued that suitable thresholds might depend on task context or dynamic parameters such as angular velocity and acceleration to be incorporated. The author group concluded that further Delphi rounds were unlikely to yield additional convergence.

### Comparison with existing literature

In terms of amplitude, previous epidemiological studies have mainly focused on time spent at higher arm elevation angles (≥60° or ≥90°), which are consistently associated with shoulder pain and long-term sickness absence (5). However, several experts in this study highlighted that such thresholds may overlook smaller, but biomechanically meaningful movements occurring below these angles. Although these smaller movements are rarely examined in epidemiological studies, clinical research shows that physiotherapists can detect shoulder abduction differences of approximately 12° under controlled conditions (38). Detection accuracy decreases as movement speed increases, supporting experts’ views that rapid small-amplitude movements are easily missed. Evidence from other joints supports this perspective, as relatively minor angular differences (5–8°) can differentiate individuals with and without pain (39), suggesting that even subtle arm movements may contribute to cumulative load despite being difficult to observe consistently.

The lack of consensus on a minimum amplitude threshold therefore reflects the challenges of balancing biomechanical relevance and observational feasibility. While prior research shows associations at higher elevation angles (5), the lower boundary at which arm movement becomes both observable and biomechanically meaningful remains uncertain. The expert responses suggest that universal lower amplitude thresholds are difficult to establish but indicate that approximately 20° represents a pragmatic limit for observable arm movements, whereas thresholds around 5° may be more suitable for sensor-based measurements.

Overall, the expert comments indicate that any operational definition of an arm movement will need to consider both the measurement method and the task context.

### Interpretation and implications

The achieved consensus definitions represent a starting point for more standardized assessment of arm exposures in occupational ergonomics. For observational practice, the time-based criteria (≤1 second for fast-paced movements and ≥4 seconds within ±5° for static postures) offer clear and operational indicators that may improve inter-rater reliability in field assessments. Although these criteria have not been validated against musculoskeletal health outcomes, they provide a structured and transparent basis for comparable exposure characterization across studies, which is a prerequisite for future validation efforts.

For research, these definitions facilitate comparability across studies and enable validation of threshold-based algorithms in sensor-based data. Although wearable sensors allow precise and continuous measurement, observational methods remain widely used in occupational risk assessment due to feasibility, cost considerations, and regulatory requirements in many workplace settings. At the same time, the increasing availability of wearable technologies is likely to expand the use of objective exposure measurements in future ergonomic research and practice. Rather than replacing observational methods, standardized operational definitions may facilitate integration between observational and technical methods, supporting gradual methodological alignment across research and practice.

Consistent with the aim of identifying operational threshold criteria, the present Delphi process focused on two parameters, amplitude and duration, as these represent core components for defining a single arm movement event. Additional parameters such as movement direction, recovery time, acceleration, and force characteristics are also biomechanically relevant. However, including multiple dynamic parameters would have substantially increased the complexity of the Delphi survey and the respondent burden. Future consensus efforts may therefore build upon the present findings by incorporating additional movement characteristics and task-specific contextual factors.

The inclusion of fast-paced arm movements was motivated by both methodological and biomechanical considerations. Exposure assessment has traditionally focused on repetition (8–10). However, repetition metrics presuppose a definition of what constitutes a single movement event. Establishing duration-based criteria therefore provides an operational basis for valid repetition metrics.

Importantly, the lack of consensus on minimal movement amplitude highlights the need for empirical calibration, for example testing which angular thresholds (eg, 10°, 20°, 50°) best correspond to observable movements and predict musculoskeletal outcomes. Future research could focus on determining whether task-specific amplitude thresholds better capture biomechanical load in different occupational contexts.

### Strengths and limitations

A key strength of this study is the participation of experts from multiple European countries, ensuring diverse professional perspectives. However, the expert panel was intentionally restricted to Europe to maintain a comparable research context and ensure feasibility. While a global panel might have broadened perspectives, it would also have complicated recruitment and introduced substantial heterogeneity in occupational environments and methodological traditions. This restriction may therefore limit the generalizability of the findings to contexts outside Europe, where ergonomic practice, work organization, and exposure assessment approaches may differ. The snowball recruitment strategy helped identify experts within this niche field. The high retention rate between rounds (88%) further supports the stability of the responses.

Of the 35 invited experts, 16 participated (46%). Compared with participants, non-participants showed a somewhat different gender distribution (47% female among non-participants versus 31% among participants), while the distribution of academic positions was broadly comparable. Although these differences were not substantial, selective participation may have influenced the perspectives represented in the consensus process and thus the resulting agreement patterns. No PhD students participated in the expert panel. All experts met predefined eligibility criteria requiring active employment in occupational research related to physical workload and arm movements.

There was a high proportion of Nordic experts (mainly from Sweden and Denmark) relative to participants from southern Europe. This geographical imbalance, combined with the absence of practitioner representatives, may introduce sampling bias and limit generalizability of the findings outside of academia and Nordic contexts. Thus, the consensus primarily reflects research-oriented perspectives, and future Delphi processes should integrate practitioner and international viewpoints to enhance external validity. Although demographic information was collected, non-respondents’ research interests were not systematically characterized, which may have influenced which items reached consensus. The decision to end the Delphi survey after two rounds reflected response stability. The lack of consensus appeared to result from differing conceptual understandings among experts, rather than from an inadequate number of Delphi iterations. Finally, as Delphi consensus reflects expert judgment rather than empirical validation, the proposed definitions should be regarded as preliminary operational definitions requiring testing under real-world conditions.

### Future research and practice

Future consensus-seeking research should build on the current findings by addressing unresolved issues, particularly the lack of agreement on minimal amplitude thresholds. Expanding future Delphi panels to include practitioners and representatives from diverse occupational and international contexts could enhance both the ecological validity and the practical relevance of the resulting definitions (40). One potential approach would be to first reach consensus among scientific experts on biomechanically meaningful movement thresholds, followed by a subsequent consensus process with practitioners to evaluate their practical feasibility in observational risk assessment. Additional Delphi rounds may also help refine task-specific thresholds that account for variations in biomechanical exposures across different job types.

Beyond further consensus efforts, research should evaluate the predictive validity of these consensus-based definitions, specifically, whether the identified thresholds for arm movements are associated with risks of neck-shoulder pain and long-term sickness absence. Such studies are needed to determine the extent to which expert-derived thresholds capture meaningful exposure-outcome relationships.

Empirical validation is likewise essential to examine how these thresholds perform under real-world conditions. Controlled observational studies and sensor-based field trials across diverse job contexts should assess inter-rater reliability, practical feasibility, and the predictive strength of combined parameters for relevant health outcomes. In practice, the implementation of such thresholds will require transparent data-processing protocols to ensure consistent exposure characterization across studies.

### Concluding remarks

This Delphi study achieved expert consensus on definitions for fast-paced arm movement (≤1 second) and static-arm posture (≥4 seconds within ±5°). No consensus was reached on what constitutes a minimum observable arm movement, as experts differed both in their views on the lower amplitude threshold and on the relevance of dynamic movement parameters. These findings show that while experts could define certain aspects of arm movement, the lower boundary for minimum movement amplitude remains uncertain and requires further empirical investigation.

## Supplementary material

Supplementary material
