# Supplementary material for: Standardizing upper arm movement definitions across observational and sensor-based methods: A Delphi consensus study among European ergonomics experts
Source: Scand J Work Environ Health. 2026 Apr 30;52(3):231–40. doi: 10.5271/sjweh.4288 (PMC13150856; doi:10.5271/sjweh.4288)
Supplement: Supplementary material [file SJWEH-52-231-S001.pdf]

Standardizing upper arm movement definitions across observational and sensor-based methods: A Delphi consensus study among European ergonomics experts[1]

by Anders Dreyer Frost, MSc,[2] Mikael Forsman, PhD, Luiz Augusto Brusaca, PhD, Andreas Holtermann, PhD, Lars Louis Andersen, PhD, Karen Søgaard, PhD, Nidhi Gupta, PhD

1. Appendices
2. Correspondence to: Anders Dreyer Frost, National Research Centre for the Working Environment, Lersø Parkallé 105, 2100 Copenhagen Ø, Denmark. [E-mail: adf@nfa.dk]

Appendix A: literature search and PubMed search identifying relevant experts for participation

Literature search: 22-02-2024

[("arm" OR "upper arm" OR "arm movement" OR "arm posture" OR "arm kinematics") AND ("accelerometer" OR "IMU" OR "inertial measurement unit" OR "inclinometry")]

Appendix B: Questionnaire sent out at the first Delphi round (R1)

Page 1 of 11

A Delphi survey on obtaining a definition of a minimum observable arm movement

Purpose of the Survey:

This survey aims to establish a consensus-based definition of a minimum observable arm movement, a fast-paced arm movement and a static arm movement.

Why?

Obtaining a consensus-based definition for minimum observable arm movement, fast-paced arm movement, and static arm movement is crucial for ensuring consistency and accuracy in research and practical applications.
It allows for standardized measurements and comparisons across studies, improving the reliability of findings and facilitating effective communication among professionals in ergonomics, occupational health, and related fields. This consensus helps to develop better guidelines for workplace safety and ergonomics interventions, ultimately improving worker health and productivity.

Survey Instructions: The questionnaire is divided into four sections: there will be more specific instructions inside the questionnaire before each of the four sections.

General Information: Questions about your age, gender, research experience, etc.

Definitions of Arm Movements: Questions focusing on the definitions of a minimum observable arm movement.

Fast-Paced Arm Movements: Questions addressing fast-paced arm movements.

Static Arm Movements: Questions pertaining to static arm movements.

General Information: If you encounter any issues, please feel free to contact us at adf@nfa.dk.

Consent Information: Your responses will be kept confidential and anonymous. All data collected will be used solely for research purposes. By proceeding with this survey, you consent to our use of your anonymized answers for our research study and the publication of results based on those answers.

We would like to give you the opportunity to be acknowledged by name in the scientific paper. Please indicate your consent to be acknowledged:

☐ Yes, I consent you to write my name in the Acknowledgment section in the scientific paper.

☐ No, I don't consent you to write my name in the Acknowledgment section in the scientific paper.

Page 2 of 11
Background information

What is your age? __________________________

What is your biological gender?

☐ Female

☐ Male

☐ Other

How many years have you been working within research?

☐ 0-5 years

☐ 6-10 years

☐ 11-15 years

☐ 16-20 years

☐ +21 years

What is your highest educational degree obtained?

☐ Bachelor/undergraduate

☐ Master's

☐ PhD

☐ Other – please state your degree _____________

Page 3 of 11
Terminology explanation:

In this survey we will use following terms, defined below. Definitions of these terms are inspired by the PEROSH report: [Assessing Arm Elevation at Work with Technical Systems - PEROSH report](#).

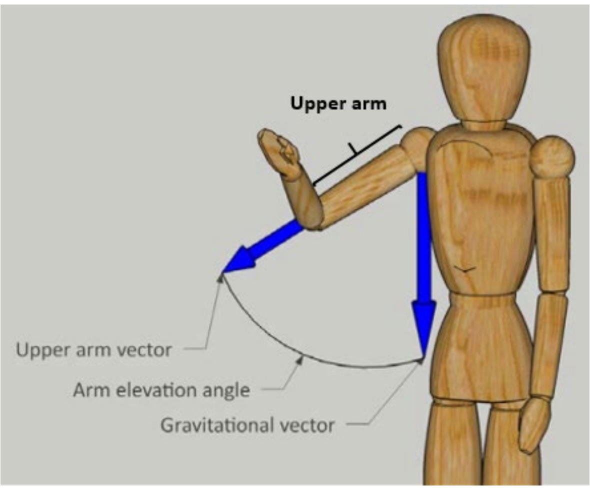

Upper arm:

We will use the term "*upper arm*" to refer to the line from the shoulder joint center (glenohumeral rotation center) to the elbow joint center (midpoint of the lateral and medial epicondyles) (Figure 1). For simplicity, we will use the term "*arm*" as a proxy for the upper arm.

Figure 1: Arm elevation angle: the angle between the upper arm vector and the vertical pointing downwards (1).

Movement (vertical movement):

"Movement" refers to the arm's motion along a vertical line. This includes both upward and downward movements. An arm movement can occur in either the coronal or sagittal planes (see Figure 2). We will measure upward and downward movements by the angle formed between the upper arm vector and the vertical line..

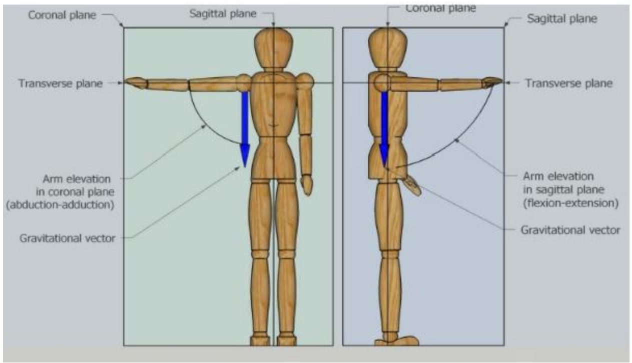

Vertical line

The vertical line is defined as the gravitational vector pointing downwards (Figure 2).

Figure 2: Arm elevation in the coronal plane (abduction-adduction) and in the sagittal plane (flexion-extension) (1).

Reference:

(1). Weber B, Douwes M, Forsman M, Könemann R, Heinrich K, Enquist H, et al. Assessing arm elevation at work with technical systems. 2018.

Page 4 of 11

Minimum observable arm movement definitions

You will now be presented for three definitions of minimum observable arm movements. For each definition, a figure and a video will be provided as a guide showing the provided arm movement definition.

Page 5 of 11

Minimum observable arm movement definitions

Below, we have provided a figure and a video illustrating examples of what we mean by the three definitions, along with explanations of the terms used.

(In the setting icon in the video you can adjust the speed of the video. Choose 0.5 to slow down the arm movement).

Definition 1: An arm movement starts each time the arm angle goes 20° higher than a previous local minimum. The next local minimum has to be at least 10° lower than a local maximum.

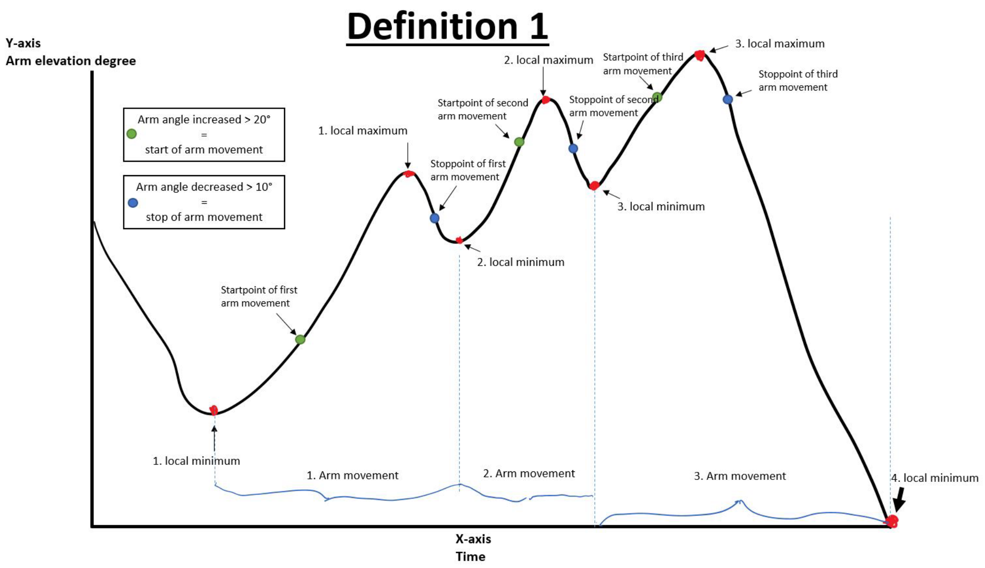

Figure 1: Figure illustrating an arm movement for definition 1

Definition 2: An arm movement starts each time the arm angle goes 20° higher than a previous local minimum. The next local minimum has to be at least 20° lower than a local maximum.

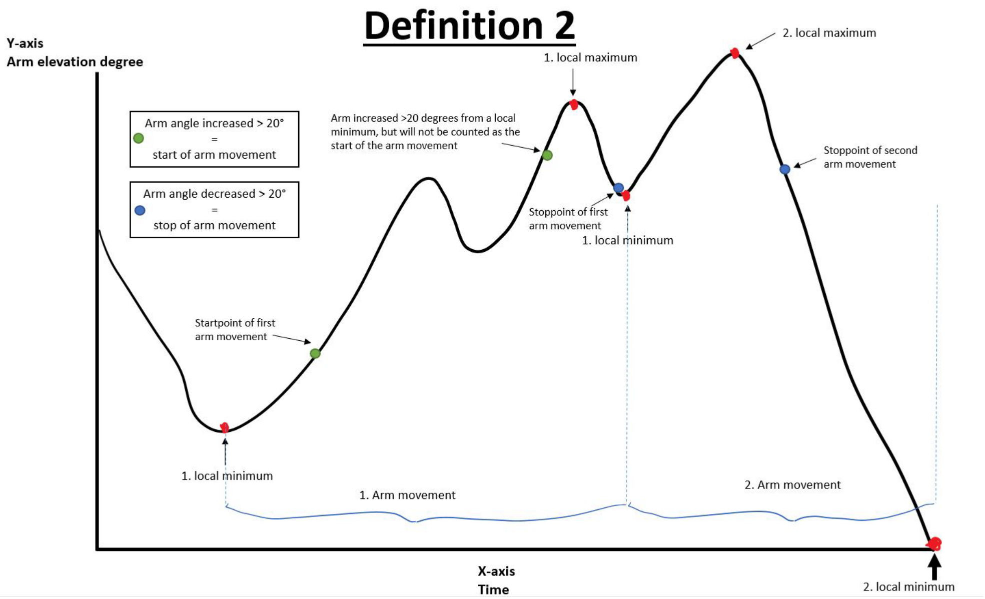

Figure 2: Figure illustrating an arm movement for definition 2

Definition 3: An arm movement starts each time the arm angle goes 50° higher than a previous local minimum. The next local minimum has to be at least 10° lower than a local maximum.

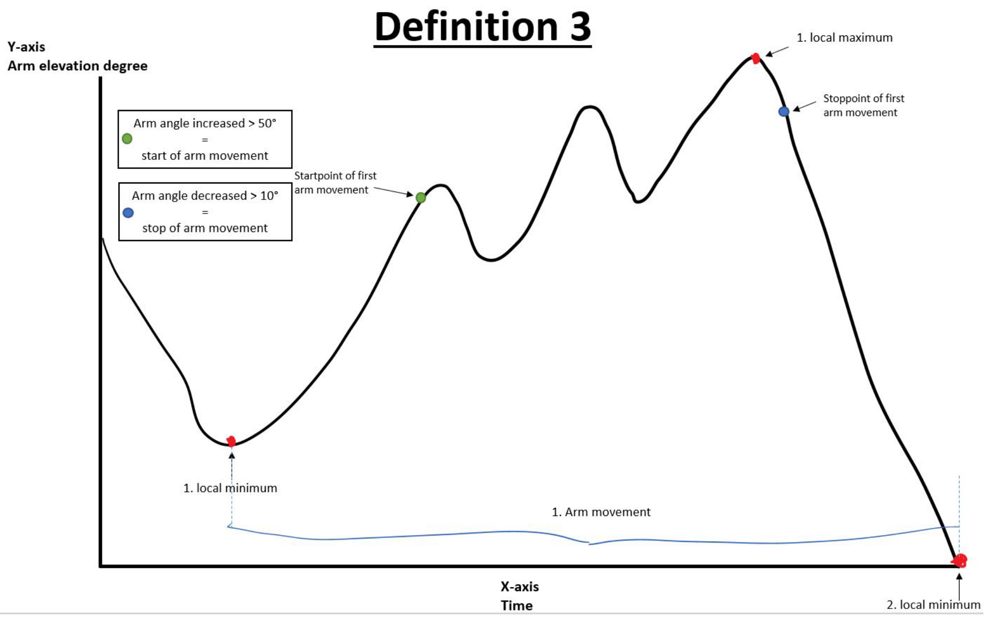

Figure 3: Figure illustrating an arm movement for definition 3

Considering the three proposed definitions of arm movements, how would you rate them based on how much you agree that each represents one arm movement?

	1) Strongly disagree	2) Disagree	3) Neither disagree or agree	4) Agree	5) Strongly agree
Definition 1: An arm movement starts each time the arm angle goes 20° higher than a previous local minimum. The next local minimum has to be at least 10° lower than a local maximum.					
Definition 2: An arm movement starts each time the arm angle goes 20° higher than a previous local					
Definition 3: An arm movement starts each time the arm angle goes 50° higher than a previous local minimum. The next local minimum has to be at least 10° lower than a local maximum.					

Also, would you explain your rating and express the reason(s) of your (dis)agreement with this definition statement?

____

Page 6 of 11

Adjusting/updating arm movement definition:

You have now been presented for three definitions of arm movements, with different start- and stop points.

Would you correct the start and/or stop of the arm movement? If yes, please write your suggested start and stop angles below, replacing the XX° (in whole degrees, fx 30).

Also, would you explain your rating and express the reason(s) of your (dis)agreement with this definition statement?

An arm movement starts each time the arm angle goes XX° higher than a previous local minimum.

____

The next local minimum has to be at least XX° lower than a local maximum. __________________

Also, would you explain your rating and express the reason(s) of your (dis)agreement with this definition statement? ________________________

Page 7 of 11

Definitions on what is "a fast-paced arm movement"?

For the next part, you will be asked to think about the duration it takes to undergo one complete cycle of an arm movement (think on the previous arm movement definitions). We will present three definitions with different duration-thresholds for when one arm movement can be categorized as a fast-paced arm movement. For each definition, a video will be provided as a guide visualizing the provided definition.

Therefore, the aim with this part of the questionnaire is to obtain a standardized/consensus based way to categorize fast-paced arm movements. All other movements not categorized as a fast-paced arm movement will then be categorized as slow/moderate-paced arm movements:

We will ask you to think about; "when can one complete cycle of one arm movement (with the previous proposed arm movement definitions in mind) be categorized as a fast-paced movement."

Definition 1: An arm movement is considered fast-paced when the whole arm movement is performed within ≤1 seconds.

Definition 2: An arm movement is considered fast-paced when the whole arm movement is performed within ≤2 seconds.

Definition 3: An arm movement is considered fast-paced when the whole arm movement is performed within ≤3 seconds.

Considering the three proposed definitions of a fast-paced arm movement, how would you rate them based on how much you agree that each represents one arm fast-paced arm movement?

	1) Strongly disagree	2) Disagree	3) Neither disagree or agree	4) Agree	5) Strongly agree
Definition 1: An arm movement is considered fast-paced when the whole arm movement is performed within ≤1 seconds.					
Definition 2: An arm movement is considered fast-paced when the whole arm movement is performed within ≤2 seconds.					
Definition 3: An arm movement is considered fast-paced when the whole arm movement is performed within ≤3 seconds.					

Also, would you explain your rating and express the reason(s) of your (dis)agreement with this definition statement? ______________________

Page 8 of 11

Adjustment/updating of a fast-paced arm movement definition:

You have now been presented for three definitions of a fast-paced arm movement, with different duration-thresholds of one arm movement.

Would you correct the duration-threshold of the arm movement? If yes, please write your suggested duration (in second(s), where the X is)

An arm movement is considered fast-paced when the whole arm movement is performed within ≤X seconds.________________________

Also, would you explain your rating and express the reason(s) of your (dis)agreement with this definition statement?_____________________

Page 9 of 11

Definitions of what is a static arm movement?

The aim of this part is to reach consensus on the time threshold for when the arm movement can be classified as a static movement. The proposed definitions below are inspired by this patent: "Van Rhijn, R.G.j.W.; Bosch, T.; Könemann, R. (http://www.google.sr/patents/EP2508127A1?cl=nl - EP2508127A1).

For each definition, a video will be provided as a guide visualizing the provided definition.

The used definition of a static posture proposed: see terminology explanation below the figure

"Subsequent peaks and local minima above a certain threshold (Ao) within an angle range of +/- A1 compared to the first of the subsequent peaks, adopted for more than T1 seconds measured from the first to the last peak within the angle range."

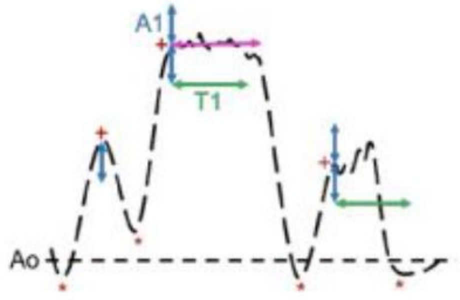

Figure 1: Simplified example of calculation of static postures

Terminology explanation:

- Ao = purple line, indicating a chosen threshold for when an arm movement has started.
- Red crosses = peak angle and local minima
- A1 = the allowed threshold for minor arm movements to not terminate the static posture.
- T1 = Duration of static posture from the first to the last peak within the angle range (green arrow).

Note: The arm degrees in the videos are indicative!

Definition 1: An upper arm is raised to a certain peak angle and maintained raised around this peak angle for ≥4 seconds (T1 threshold). Minor movements (A1) of the arm (e.g. +/- ≤5°, A1 threshold) will not terminate the static posture.

Definition 2: An upper arm is raised to a certain peak angle and maintained raised around this peak angle for ≥4seconds (T1 threshold). Minor movements (A1) of the arm (e.g. +/- ≤10°, A1 threshold) will not terminate the static posture.

Definition 3: An upper arm is raised to a certain peak angle and maintained raised around this peak angle for ≥4seconds (T1 threshold). Minor movements (A1) of the arm (e.g. +/- ≤15°, A1 threshold) will not terminate the static posture.

Now you have seen the three pre-defined definitions on what could be considered as a static arm movement.

We would like to know your assessment of these three definitions.

Considering the three proposed definitions of a static arm movement, how would you rate them based on how much you agree that each represents one static arm movement?

1) Strongly disagree 2) Disagree 3) Neither disagree or agree4) Agree 5) Strongly agree

Definition 1: An upper arm is raised to a certain peak angle and maintained raised around this peak angle for ≥4 seconds (T1 threshold). Minor movements (A1) of the arm (e.g. +/- ≤5°, A1 threshold) will not terminate the static posture.

Definition 2: An upper arm is raised to a certain peak angle and maintained raised around this peak angle for ≥4seconds (T1 threshold). Minor movements (A1) of the arm (e.g. +/- ≤10°, A1 threshold) will not terminate the static posture.

Definition 3: An upper arm is raised to a certain peak angle and maintained raised around this peak angle for ≥4seconds (T1 threshold). Minor movements (A1) of the arm (e.g. +/- ≤15°, A1 threshold) will not terminate the static posture.

Also, would you explain your rating and express the reason(s) of your (dis)agreement with this definition statement?______________________

Page 10 of 11

Adjustment/updating of a static arm movement definition:

You have now been presented for three definitions of static arm movements, with different durations and minor movement thresholds.

Would you correct the duration and/or the minor movement threshold of the arm movement, to be considered as a static arm movement? If yes, please write your suggested duration (in second(s), and/or degrees where the X is)

An upper arm is raised to a certain peak angle and maintained raised around this peak angle for ≥X seconds.______________________

Minor movements of the arm (e.g. +/- ≤X°, A1 threshold) will not terminate the static posture.________________________

Minor movements of the arm (e.g. +/- ≤X°, A1 threshold) will not terminate the static posture

____

Page 11 of 11

You have now reached the end of the questionnaire.

Thank you for taking your time to complete the questionnaire. Your inputs are valuable for us.

We aim to send out the new and revised qustionnaire with a short report with summarized answers from this round in end September/start October.

If you have any additional comments please send an email to me, Anders at e-mail adf@nfa.dk.

Appendix C: Questionnaire sent out at the second Delphi round

Page 1 of 8

A Delphi Survey on Defining an Arm Movement - Round 2

Purpose of the Delphi survey:

The overall aim of this survey is to establish a consensus-based definition for the following:

A minimum observable arm movement

A fast-paced arm movement

A static arm movement.

Following Delphi Round 1, consensus was reached on the definitions of fast-paced arm movement and static arm movement. Therefore, questions related to these aspects are not included in Delphi Round 2.

The primary goal of this survey is to reach a consensus on the following definitions:

1) a consensus definition of what is one minimum observable arm movement.

   a) pros and cons of this definition.

2) a consensus definition of what is one arm movement using wearables.

   a) pros and cons of this definition.

These definitions aim to guide accelerometer research, ensuring standardized and consistent interpretation of findings.

Survey instructions: In this round, you will be asked to assess two main definitions.

A minimum observable arm movements: Questions focusing on the definitions of a minimum observable arm movement.

An arm movements using wearables: Questions focusing on the definitions of an arm movement using wearables.

Furthermore, what is a fast-paced arm movement using wearable data?

General Information:

If you encounter any issues, please feel free to contact us at adf@nfa.dk.

Consent Information:

Your responses will be kept confidential and anonymous. All data collected will be used solely for research purposes. By proceeding with this survey, you consent to our use of your anonymized answers for our research study and the publication of results based on those answers.

Page 2 of 8

Terminology explanation

In this survey we will use following terms, defined below. Definitions of these terms are inspired by the PEROSH report: Assessing Arm Elevation at Work with Technical Systems - PEROSH report.

Upper arm:

We will use the term "upper arm" to refer to the line from the shoulder joint center (glenohumeral rotation center) to the elbow joint center (midpoint of the lateral and medial epicondyles) (Figure 1). For simplicity, we will use the term "arm" as a proxy for the upper arm.

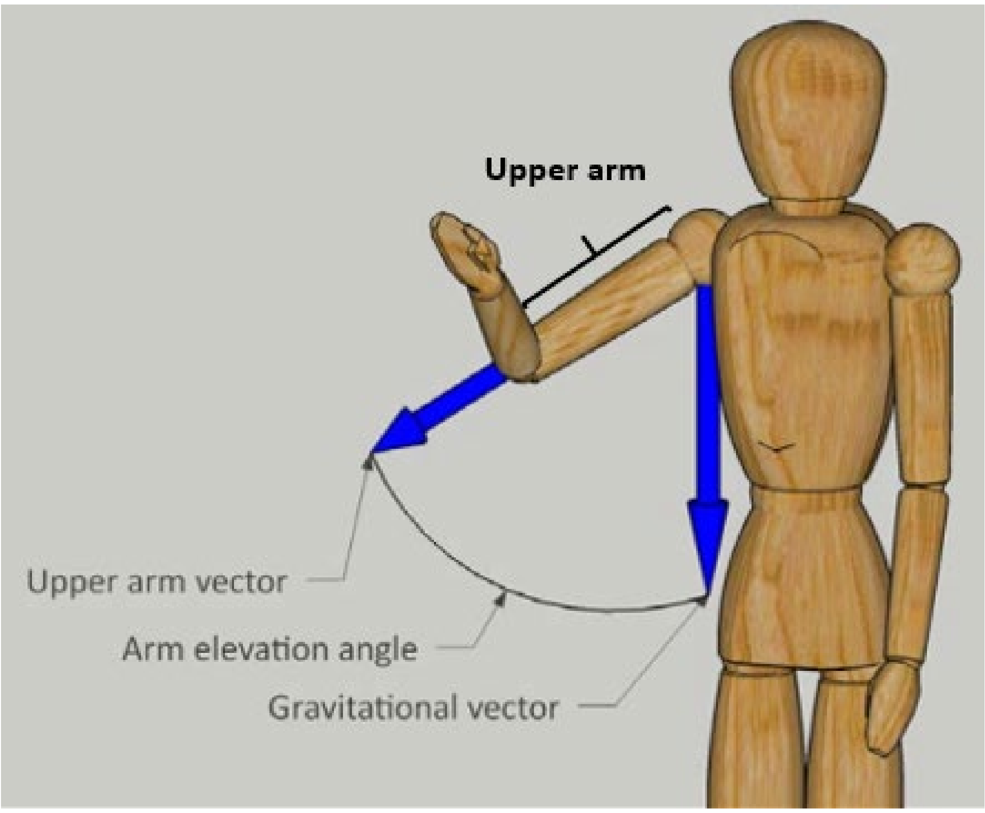

Figure 1: Arm elevation angle: the angle between the upper arm vector and the vertical pointing downwards (1).

Movement (vertical movement):

"Movement" refers to the arm's motion along a vertical line. This includes both upward and downward movements. An arm movement can occur in either the coronal or sagittal planes (see Figure 2). We will measure upward and downward movements by the angle formed between the upper arm vector and the vertical line..

Vertical line

The vertical line is defined as the gravitational vector pointing downwards (Figure 2).

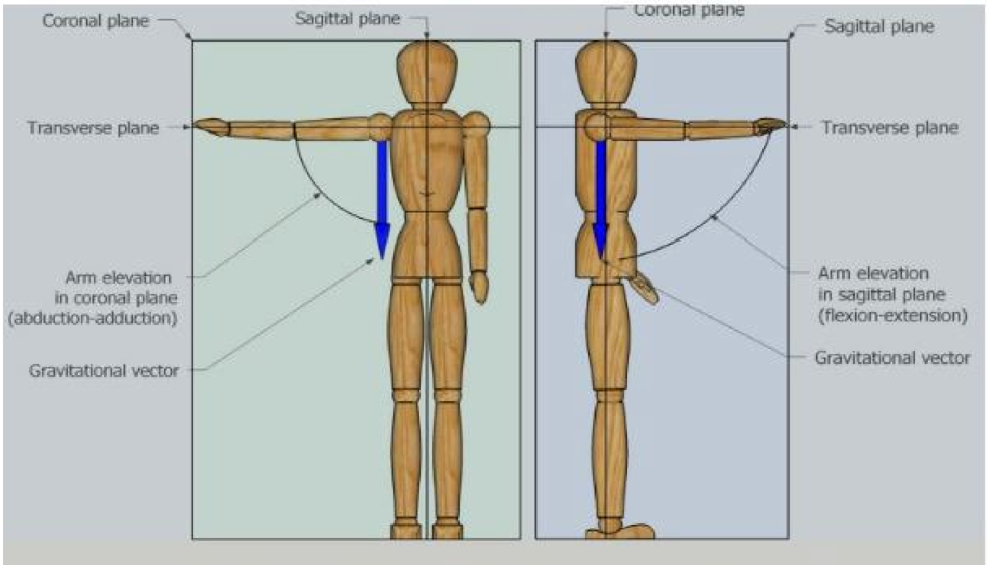

Figure 2: Arm elevation in the coronal plane (abduction-adduction) and in the sagittal plane (flexion-extension) (1).

Reference:

(1). Weber B, Douwes M, Forsman M, Könemann R, Heinrich K, Enquist H, et al. Assessing arm elevation at work with technical systems. 2018.

Page 3 of 8

Rate the following definition: A minimum observable arm movement

Definition: A minimum observable arm movement (see Figure for details)

"An upper arm movement starts at a local minimum and is counted when the upper arm has gone upwards by more than 20°, and ending when the upper arm has gone down by more than 20° from a local maximum and reaches a second local minimum."

Question:

Do you agree that this definition represents a minimum observable arm movement?

1) Strongly disagree 2) Disagree 3) Neither disagree or agree4) Agree 5) Strongly agree

Figure: Arm Movement Detection Model

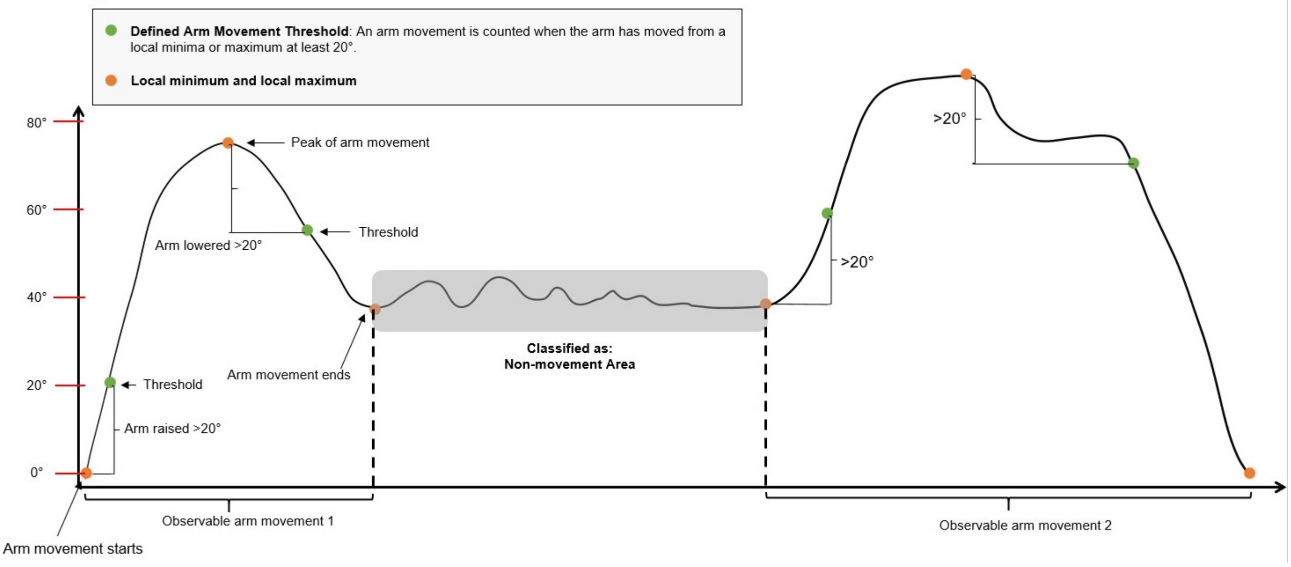

Would you explain your rating and express the reason(s) of your (dis)agreement with this definition statement?_____________________________

Page 4 of 8

Adjusting/updating the minimum observable arm movement definition:

Based on the definition you was presented for, would you suggest other start- and stop points?

If yes, please write your suggested start and stop angles below, replacing the XX° (in whole degrees, fx 10).

Also, would you explain your rating and express the reason(s) of your (dis)agreement with this definition statement?

An upper arm movement starts at a local minimum and is counted when the upper arm has gone upwards by more than XX°._____________________

And ending when the upper arm has gone down by more than XX° from a local maximum and reaches a second local minimum.___________________

Page 5 of 8

The Need for Standardizing Arm Movement Definitions in Wearable Research

With the increasing use of accelerometers and other wearable devices in research, it is crucial to establish consistent methods for using and interpreting the data. Variability in how arm movements are defined and measured can lead to inconsistencies in research outcomes, making it difficult to compare findings across studies.

To address this, we aim to develop a consensus definition of what constitutes one arm movement when using data from wearables, in the next section of the questionnaire. By standardizing this

definition, we can enhance the reliability and comparability of research that uses wearable devices to study arm movements.

In the following section, you will be asked to evaluate a proposed definition of one arm movement using wearables.

Page 6 of 8

Rate the following adjusted definition on: An arm movement (using wearables)

Definition: An arm movement using wearables (see Figure for details):

"An upper arm movement starts at a local minimum and is counted when the upper arm has gone upwards by more than 5°, and ending when the upper arm has gone down by more than 5° from a local maximum and reaches a second local minimum."

Question:

Do you agree that this definition accurately represents one arm movement using wearables?

1) Strongly disagree 2) Disagree 3) Neither disagree or agree4) Agree 5) Strongly agree

Figure: Arm Movement Detection Model

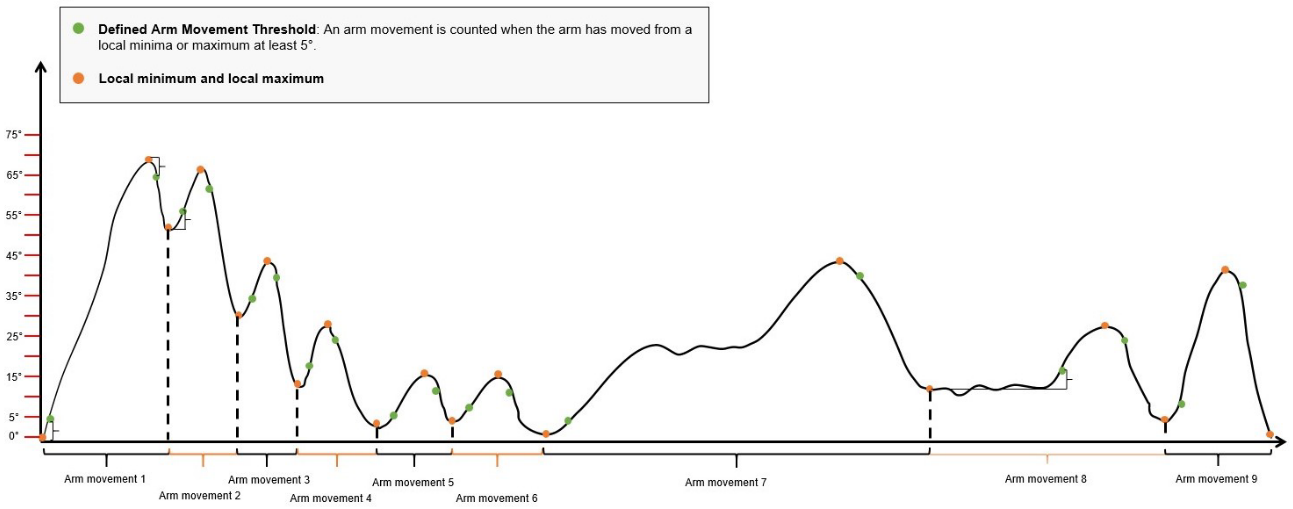

Would you explain your rating and express the reason(s) of your (dis)agreement with this definition statement?________________________

Page 7 of 8

Adjusting/updating the arm movement definition:

Based on the definition you were presented with, would you suggest other start and stop points?

If yes, please write your suggested start and stop angles below, replacing the XX° (in whole degrees, e.g., 10).

Also, could you explain your rating and express the reason(s) for your agreement or disagreement with this definition?

An upper arm movement starts at a local minimum and is counted when the upper arm has gone upwards by more than XX°.__________________

And ending when the upper arm has gone down by more than XX° from a local maximum and reaches a second local minimum.__________________

Page 8 of 8

You have now reached the end of the questionnaire.

Thank you for taking your time to complete the questionnaire. Your inputs are valuable for us.

We aim to send out the new and revised qustionnaire with a short report with summarized answers from this round in end September/start October.

If you have any additional comments please send an email to me, Anders at e-mail adf@nfa.dk.
